# Hydrogen-Rich Saline Attenuates Acute Renal Injury in Sodium Taurocholate-Induced Severe Acute Pancreatitis by Inhibiting ROS and NF-*κ*B Pathway

**DOI:** 10.1155/2015/685043

**Published:** 2015-03-23

**Authors:** Qiao Shi, Kang-Shu Liao, Kai-Liang Zhao, Wei-Xing Wang, Teng Zuo, Wen-Hong Deng, Chen Chen, Jia Yu, Wen-Yi Guo, Xiao-Bo He, Ablikim Abliz, Peng Wang, Liang Zhao

**Affiliations:** ^1^Department of General Surgery, Renmin Hospital of Wuhan University, Wuhan, Hubei 430060, China; ^2^Department of Hepatobiliary Surgery, Central Hospital of Enshi Autonomous Prefecture, Enshi, Hubei 445000, China

## Abstract

Hydrogen (H_2_), a new antioxidant, was reported to reduce ^•^OH and ONOO^−^ selectively and inhibit certain proinflammatory mediators to product, without disturbing metabolic redox reactions or ROS involved in cell signaling. We herein aim to explore its protective effects on acute renal injury in sodium taurocholate-induced acute pancreatitis and its possible mechanisms. Rats were injected with hydrogen-rich saline (HRS group) or normal saline (SO and SAP group) through tail intravenously (6 mL/kg) and compensated subcutaneously (20 mL/kg) after successful modeling. Results showed that hydrogen-rich saline attenuated the following: (1) serum Cr and BUN, (2) pancreatic and renal pathological injuries, (3) renal MDA, (4) renal MPO, (5) serum IL-1*β*, IL-6, and renal TNF-*α*, HMGB1, and (6) tyrosine nitration, I*κ*B degradation, and NF-*κ*B activation in renal tissues. In addition, it increased the level of IL-10 and SOD activity in renal tissues. These results proved that hydrogen-rich saline attenuates acute renal injury in sodium taurocholate-induced acute pancreatitis, presumably because of its detoxification activity against excessive ROS, and inhibits the activation of NF-*κ*B by affecting I*κ*B nitration and degradation. Our findings highlight the potential value of hydrogen-rich saline as a new therapeutic method on acute renal injury in severe acute pancreatitis clinically.

## 1. Introduction

Acute pancreatitis (AP) is an acute inflammatory disorder with variable severity ranging from self-limited to severe conditions. It is about 20% of AP experience severe attacks, such as complicate with multiple-organ dysfunction syndrome frequently [1]. Acute renal failure (ARF) is a common complication of severe acute pancreatitis (SAP); the morbidity of ARF in SAP was 14% to 43%, and its mortality was 71% to 84% [2]. Usually, acute renal failure is a major prognostic factor in SAP and is known to occur in the early stage of this disease. It could promote the failure of other organ systems and accelerate the progression of the disease, subsequently leading to death.

Although there are good evidences illustrating that “autodigestion” of pancreatic gland is the main cause of the disease, the exact mechanism of AP remains entirely unclear. An available study showed activated enzymes and oxygen-free radicals injure cells, tissues, and organs and cause the release of cytokines and vasoactive mediators [3]. Acute renal injury in SAP is closely related to oxidative stress, the activation, and release of various cytokines and inflammatory mediators, including nuclear factor kappa B (NF-*κ*B), tumor necrosis factor (TNF-*α*), interleukin (IL)-1*β*, interleukin IL-6, interleukin IL-10, and high mobility group box protein 1 (HMGB1) [4, 5]. Thence, reducing oxidative stress and proinflammatory mediators might be a good therapeutic strategy to attenuate acute renal injury in SAP.

Hydrogen gas (H_2_), a well-known molecule with the simplest structure, has been demonstrated recently to have selectively reduced ROS and anti-inflammatory properties [6, 7]. Instead of H_2_, hydrogen-rich saline, which is safer and easier for administration, may be more suitable for clinical applications. Effects of molecular hydrogen have been observed essentially in ischemia-reperfusion (I/R) injury [7, 8], lipid, and glucosemetabolism in patients with type 2 diabetes impaired glucose tolerance [9], Alzheimer's and Parkinson's disease [10, 11], kidney transplantation [12], acute pancreatitis [13–15], and other oxidative stress-related diseases. However, there are no experimental studies about extrapancreatic organ injuries and few researches to explore the protective mechanisms of hydrogen-rich saline concretely for SAP. Therefore, we designed this experiment to evaluate the effects of hydrogen-rich saline on acute renal injury in SAP and explore its possible mechanisms.

## 2. Materials and Methods

### 2.1. Animals

Male SPF Wistar rats, with weight from 200 g to 250 g, were obtained from the Center of Experimental Animals of Hubei Academy of Medical Sciences, Wuhan, China. All animals were housed under standardized conditions with a 12-hour day-night rhythm with free access to standard laboratory chow and water. The study was approved by the Ethics Committee of Wuhan University and performed in accordance with the EC regulations (Official Journal of European Community L385 12/18/1986) and the NIH standards (Guide for the Care and Use of Laboratory Animal, National Institutes of Health's publication 85-23, revised 1996).

### 2.2. Preparation of Hydrogen-Rich Saline

Hydrogen-rich saline was generously offered by Professor Xuejun Sun (the Second Military Medical University, Shanghai, China) and prepared to confirm the content of hydrogen in saline as described previously [16]. In short, H_2_ gas (0.4 mPa) was dissolved in normal saline for at least 2 hours to reach supersaturated level (>0.6 mmol/L). The hydrogen-rich saline was stored under atmospheric pressure at 4°C in an aluminum bag with no dead volume and sterilized by gamma radiation before use.

### 2.3. Experimental Model and Groups

Rats were fasted overnight and given fresh tap water ad libitum. Anesthesia was administered by intraperitoneal injection of 10% chloraldurat (0.3 mL/100 g). The SAP model was induced by a standard retrograde infusion of a freshly prepared 5% sodium taurocholate solution (Sigma-Aldrich, St Louis, Mo, 0.1 mL/100 g) into the biliopancreatic duct after laparotomy. Equivalent volume of normal saline solution was substituted for 5% sodium taurocholate solution in the sham-operation control group. The incision was closed with a continuous 3-0-silk suture, and 2 mL/100 g of saline was injected into the back subcutaneously to compensate for the fluid loss. 72 rats were randomly divided into three groups: (1) hydrogen-rich saline treatment group (HRS group, *n* = 24) where the rats were administered as a tail intravenously (0.6 mL/100 g) and compensated subcutaneously (2 mL/100 g) with hydrogen-rich saline at 5 min after successful SAP modeling; (2) severe acute pancreatitis model group (SAP group, *n* = 24) where these rats received an equivalent volume of the normal saline instead of hydrogen-rich saline; (3) Sham operation group (SO group, control, *n* = 24) where these rats received an equivalent volume of the normal saline after successful sham-operation. All the rats were sacrificed at 3, 12, and 24 hours after the operation (*n* = 8 per group).

### 2.4. Blood and Tissue Preparation

For each group of the studies, rats were sacrificed by taking blood via intracardiac puncture at respective time points. Blood samples were collected for centrifuging, and serum was stored at −20°C. Pancreas and kidney were harvested and fixed in 4% phosphate-buffered formaldehyde for histopathology observation. The remaining parts of the pancreatic and kidney tissues were immediately frozen in liquid nitrogen and stored at −80°C for assay.

### 2.5. Serum AMY, LIP, BUN, and Cr Assays

Blood samples were centrifuged at 3000 rpm for 10 min and kept at −20°C until assays. Amylase (AMY), lipase (LIP), creatinine (Cr), and urea nitrogen (UNB) were measured to use standard procedure with a full automatic chemistry analyzer (Olympus AU 2700 Analyzer, Olympus Inc, Tokyo, Japan).

### 2.6. Histological Examination

For histological analysis, formaldehyde-fixed specimens were embedded in paraffin, sectioned at 4 um thick, and treated with hematoxylin-eosin staining. Sections were evaluated under light microscope (Olympus Optical Ltd., Tokyo, Japan) by two independent pathologists who were blinded to this experiment. Scoring of the severity of pancreatitis was based on edema, inflammation, vacuolization, and necrosis according to the scale described by Schmidt et al. [17]. Evaluation of kidney injury was quantified by using a histopathological score, as outlined by Paller et al. [18]. For each kidney 100 cortical tubules from at least 10 different areas were scored, and care was taken to avoid repeated scoring of different convolutions of the same tubule. Higher scores represented more severe damage (maximum score per tubule was 10), with points given for the presence and extent of tubular epithelial cell flattening (1 point), brush border loss (1 point), cell membrane bleb formation (1 or 2 points), interstitial edema (1 point), cytoplasmic vacuolization (1 point), cell necrosis (1 or 2 points), and tubular lumen obstruction (1 or 2 points).

### 2.7. Renal MDA and SOD Assays

Tissue MDA level, a marker of lipid peroxidation, was measured using a commercial MDA assay kit (Nanjing Jiancheng Bioengineering Institute, Nanjing, China). Briefly, hydroxytoluene combined with thiobarbituric acid was able to become red. The absorbance of condensation products was tested at a wavelength of 532 nm. The levels of MDA in renal tissue were normalized against total protein (mg protein/mL). The activity of superoxide dismutase (SOD) in renal tissues was measured using a commercial assay kit (Nanjing Jiancheng Bioengineering Institute, Nanjing, China), following the manufacturer's instructions. Briefly, this assay kit uses a thiazole salt for detection of superoxide anions to produce a colored product; absorbance was tested at a wavelength of 450 nm. One unit (U) of SOD was defined as the sum of enzyme needed to produce 50% dismutation of superoxide radical. The total tissue protein concentration was measured by a commercial kit (Beyotime Institute of Biotechnology, Shanghai, China), and the activity of SOD was expressed as units of enzyme activity of SOD per milligram of protein (U/mg protein).

### 2.8. Renal MPO Activity Assay

Myeloperoxidase (MPO), an indicator of neutrophil infiltration into the renal parenchyma, was measured as described previously [19]. Myeloperoxidase activity was measured photometrically with 3,3′,5′,5-tetramethylbenzidine as a substrate, and the reaction happened by adding hydrogen peroxide to the medium. Renal cortical samples were weighed and homogenized in 1 : 19 (wt/vol) in ice-cold buffer. Myeloperoxidase activity assay was performed with a commercial kit (Nanjing Jiancheng Bioengineering Institute, Nanjing, China). Absorbance was measured at a wavelength of 460 nm. The results were expressed as units of enzyme activity of MPO per gram of wet tissue (U/g wet tissue).

### 2.9. Immunohistochemistry for NF-*κ*B and 3-Nitrotyrosine in Renal Sections

The expressions of NF-*κ*B p65 and 3-nitrotyrosine were detected by immunohistochemistry in renal tissues. Briefly, the renal sections (4 um) were dewaxed and incubated with 3% H_2_O_2_ in methanol at 37°C for 10 min to quench endogenous peroxidase activity. Following a 20 min blocking step with 5% normal goat serum diluted in 0.01% PBS, the primary antibodies of rabbit monoclonal NF-*κ*B p65 (1 : 100, abcam, UK) and mouse monoclonal 3-nitrotyrosine (1 : 150, abcam, UK) were applied and incubated for 2 h in a moisture chamber at 37°C. Sections were counterstained with hematoxylin. The dark reddish-brown areas were the NF-*κ*B p65 and 3-nitrotyrosine-containing cells. The positive staining cells were observed under microscope (400x) and evaluated by two pathologists in a blind manner.

### 2.10. Cytokines Assay

Serum concentrations of IL-1*β* and IL-6 were measured by the commercially available enzyme-linked immunosorbent assay (ELISA), according to the manufacturer's instructions (R&D Systems, Minneapolis, Minn.). The absorbance was read on an automated ELISA reader and concentrations were calculated according to the standard curve run on each assay plate. All samples were assayed 3 times.

### 2.11. Western Blot Analysis

Renal NF-*κ*B p65, I*κ*B*α*, TNF-*α*, and IL-10 at 12 hours and HMGB1 at 24 hours after successful modeling were measured by Western blot analysis. Cytoplasmic and nuclear proteins were extracted using the nuclear-cytosol extraction kit (Beyotime Institute of Biotechnology, Shanghai, China), following manufacturer's instructions. Proteins were evaluated by the Bradford method with bovine serum albumin as a standard. Briefly, equal sums of protein samples were separated on 8% or 10% sodium dodecyl sulphate polyacrylamide (SDS-PAGE) gels and then transferred to a nitrocellulose membrane. Membranes were blocked with blocking buffer (TBS containing 5% nonfat dry milk, 0.1% Tween-20) for 2 hours at room temperature. The nuclear proteins were incubated with primary antibodies of rabbit monoclonal anti-rat NF-*κ*B p65 antibody (1 : 1000, Abcam, UK) and anti-Lamin B1 antibody (1 : 1000, Cell Signaling Technology, USA). Meanwhile, the cytoplasmic proteins were incubated with primary antibodies of rabbit monoclonal anti-rat I*κ*B*α* antibody (1 : 1000, Abcam, UK) and *β*-actin antibody (1 : 1000, Abcam, UK). In addition, the total proteins were incubated with primary antibodies of rabbit monoclonal anti-rat TNF-*α* antibody (1 : 1000, Abcam, UK), IL-10 antibody (1 : 1000, Abcam, UK) and HMGB1 (1 : 1000, Cell Signaling Technology, USA), and *β*-actin antibody (1 : 1000, Abcam, UK) overnight at 4°C. The membranes were washed with TBST (TBS containing 0.05% Tween-20) and then incubated with horseradish peroxidase-conjugated goat anti-rabbit secondary antibodies (1 : 5000, Pierce Biotechnology, Rockford, ш) for 1 h at room temperature and developed with the use of ECL reagent (Millipore, Bedford, Mass) and captured on light-sensitive imaging film (Kodak). The protein bands were quantified with densitometry (Quantity One 4.5.0 software; Bio-Rad Laboratories, Richmond, Calif.).

### 2.12. Statistical Analysis

All data were expressed as means ± standard deviation values. Data were compared between all groups by one-way analysis of variance (ANOVA). Statistical analysis was performed with the SPSS statistical package (SPSS 17.0, Chicago, III). A value of *P* < 0.05 was regarded as a significant difference.

## 3. Result

### 3.1. Serum AMY, LIP, Cr, and BUN Assay

**Figure 1:**
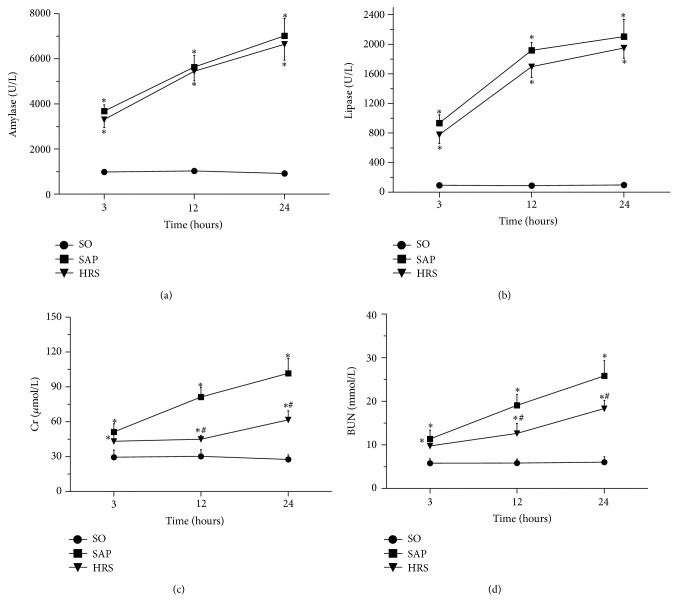
Serum levels of AMY, LIP, Cr, and BUN in all groups of rats. (a) Serum AMY level, (b) serum LIP level, (c) serum Cr level, and (d) serum BUN level. Results are expressed as mean (SD), 8 rats per group.^*^ *P* < 0.05 versus respective SO group; ^#^ *P* < 0.05 versus respective SAP group.

Compared with SO group, serum AMY and LIP levels were significantly increased in the SAP group at respective time points (*P* < 0.05), but there were no significant differences between SAP group and HRS group (*P* > 0.05). SAP group had a significant increase on the levels of serum Cr and BUN (*P* < 0.05). Hydrogen-rich saline induced a significant decrease on the levels of serum Cr and BUN at 12 and 24 hours after SAP (*P* < 0.05) (Figure 1).

### 3.2. Pancreatic and Renal Histological Analysis and Scores

**Figure 2:**
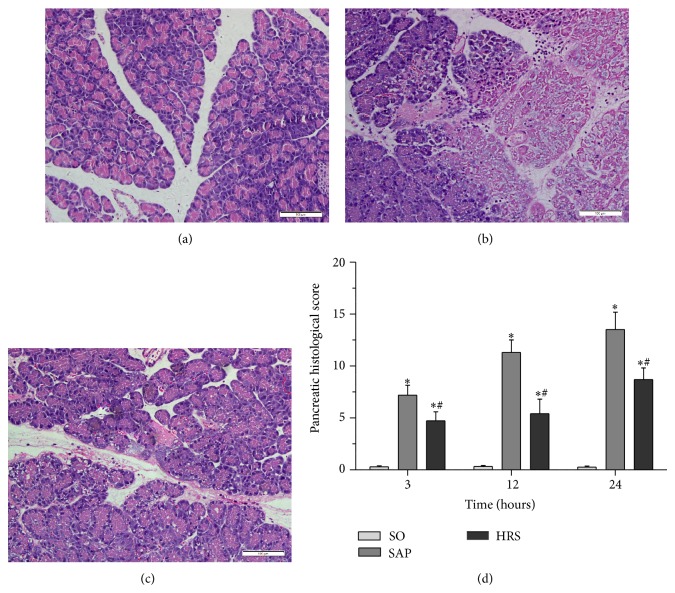
Morphologic changes of pancreas at 12 hours and the pancreatic histological scores at each time point. (a) SO group, (b) SAP group, (c) HRS group, and (d) pathological scores of pancreas at each time point. ^*^ *P* < 0.05 versus SO group; ^#^ *P* < 0.05 versus SAP group. The figure is representative of at least 3 experiments performed on different experimental days (original magnification ×200).

**Figure 3:**
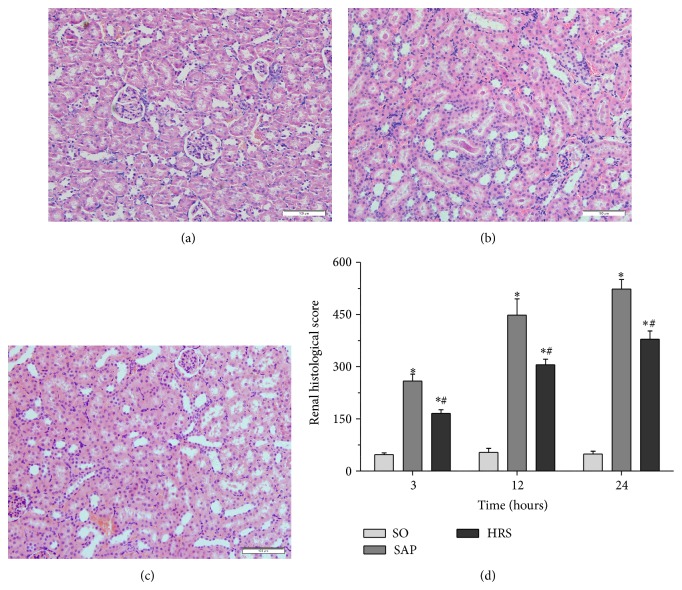
Morphologic changes of the kidney at 12 hours and the renal histological scores at each time point. (a) SO group, (b) SAP Group, (c) HRS group, and (d) the pathological scores of the kidney at each time points. ^*^ *P* < 0.05 versus SO group; ^#^ *P* < 0.05 versus SAP group. The figure is representative of at least 3 experiments performed on different experimental days (original magnification ×200).

Representative histological sections are shown in Figures 2(a)–2(c) (pancreas) and Figures 3(a)–3(c) (kidney) at 12 hours. The normal histological structure of pancreas was observed in SO group (Figure 2(a)). Conspicuous pancreatic edema, interstitial leukocyte infiltration, intrapancreatic hemorrhage, and necrosis were observed in the SAP group (Figure 2(b)). Compared with SAP group, the extent and severity of the pancreatic histological injuries were significantly reduced in the HRS group (*P* < 0.05, Figure 2(c)). As shown in Figure 2(d), all time points of the pancreatic histological scores in the HRS group were significantly more decreased than SAP group (*P* < 0.05).

SO group represents normal histological structure of renal glomerulus, tubule, and interstitium (Figure 3(a)). In contrast, SAP group at 12 hours demonstrated the identifiable features of kidney injury including typical histological signs of glomerular and tubular damage. Degeneration of the glomerulus, blurred cellular boundaries, tubular epithelial cells swelling and necrosis, interstitial hemorrhage, tubular lumen stenosis, a large amount of tubular casts formatting, and the presence of inflammatory cell infiltration were observed (Figure 3(b)). Kidneys obtained from rats that were treated with hydrogen-rich saline were demonstrated to have milder histological features and lower pathological scores when compared with SAP group (Figure 3(c)). As shown in Figure 3(d), the renal histological scores in HRS group were significantly decreased when compared with SAP group in the corresponding time points (*P* < 0.05).

### 3.3. Hydrogen-Rich Saline Treatment Reduced Oxidative Stress and Renal Neutrophil Infiltration

**Figure 4:**
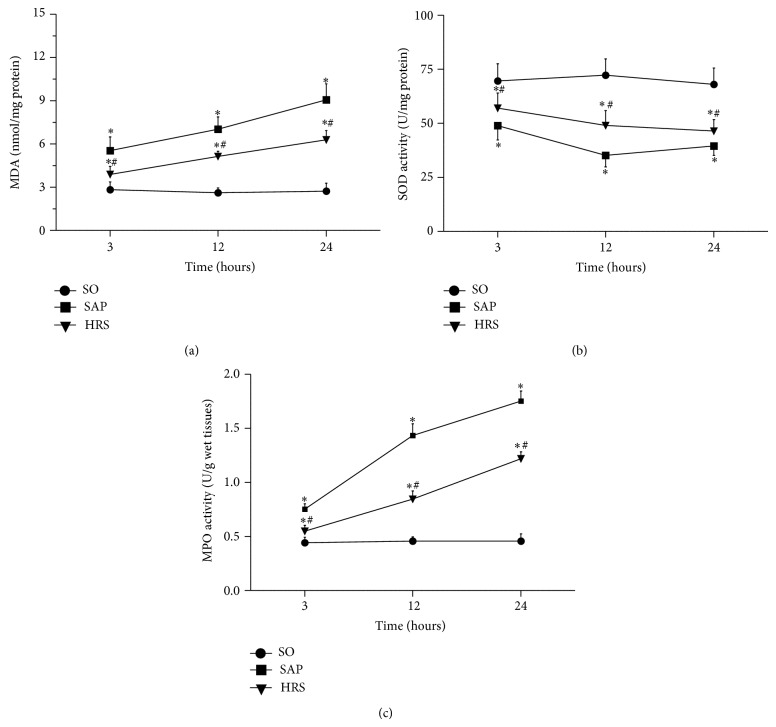
Effects of hydrogen-rich saline reduced oxidative stress and renal neutrophil infiltration in renal tissues. (a) Content of MDA, (b) SOD activity, and (c) MPO activity. Results are expressed as mean (SD), 8 rats per group. ^*^ *P* < 0.05 versus respective SO group; ^#^ *P* < 0.05 versus respective SAP group.

Although enhanced oxidative stress was observed in all acute pancreatitis animals, as evidenced by elevated renal tissue MDA level, the elevation appeared to be significantly inhibited by hydrogen-rich saline treatment (*P* < 0.05, Figure 4(a)). Moreover, similar changes were observed for SOD activity in renal tissues. This was found to be significantly depleted in acute pancreatitis rats, presumably as a result of oxidative stress processes. In contrast, treatment with hydrogen-rich saline effectively improved the activity of SOD in kidney (*P* < 0.05, Figure 4(b)). The infiltration of neutrophils into the kidneys was evaluated by renal MPO activity. Myeloperoxidase activity was significantly elevated after SAP at each time point compared with SO group (*P* < 0.05). However, HRS group had a significant reduced when compared with SAP group (*P* < 0.05, Figure 4(c)).

### 3.4. Immunohistochemistry Analysis for NF-*κ*B p65 and 3-Nitrotyrosine

**Figure 5:**
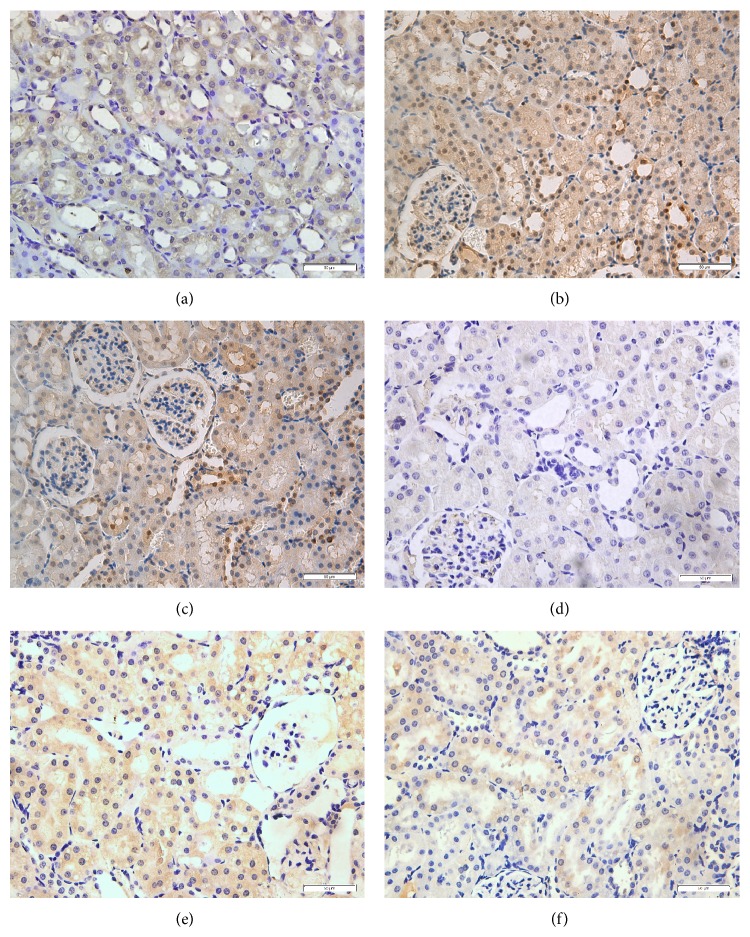
The expressions of NF-*κ*B p65 and 3-nitrotyrosine were detected in renal tissues at 12 hours in all groups (original magnification ×400). (a) In SO group, weak immunoreactivity of NF-*κ*B was mainly shown in cytoplasts. (b) In SAP group, intense immunoreactivity of NF-*κ*B was shown in nucleus. (c) In HRS group, lower immunoreactivity of NF-*κ*B was shown in nucleus than SAP group. (d) In SO group, exceedingly weak immunoreactivity of 3-nitrotyrosine was shown in cytoplasts. (e) In SAP group, intense immunoreactivity of 3-nitrotyrosine was shown in cytoplasts. (f) In HRS group, lower immunoreactivity of 3-nitrotyrosine was shown in cytoplasts than SAP group.

Immunohistochemical assay was used to evaluate the expressions of NF-*κ*B p65 and 3-nitrotyrosine in renal tissues. In SO group, the weak expressions of NF-*κ*B P65 was concentrated in the cytoplasms (Figure 5(a)); the expressions of 3-nitrotyrosine were exceedingly weak in cytoplasm (Figure 5(d)). In SAP group, intense immunoreactivity of NF-*κ*B p65 was expressed in nucleus (Figure 5(b)) and intense immunoreactivity of 3-nitrotyrosine was concentrated in cytoplasts (Figure 5(e)). In HRS group, the expressions of NF-*κ*B p65 have a significant decrease in nucleus (Figure 5(c)), the expressions of 3-nitrotyrosine have a significant decrease in cytoplasms (Figure 5(f)).

### 3.5. Hydrogen-Rich Saline Treatment Reduced the Production of Proinflammatory Cytokines

**Figure 6:**
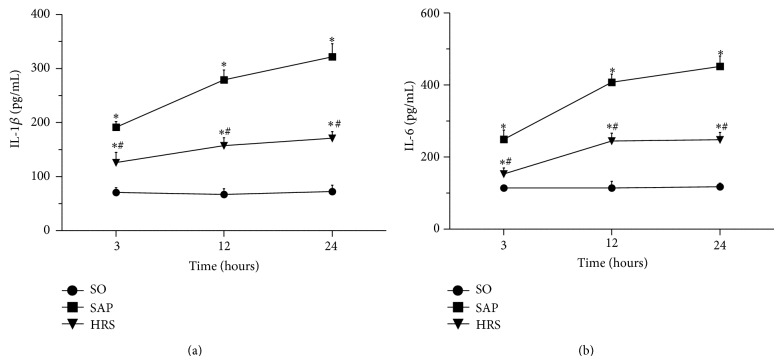
Effects of hydrogen-rich saline reduced proinflammatory cytokines. Serum levels of IL-1*β* and IL-6 were quantified by ELISA at different time points after SAP. (a) Serum IL-1*β* Level, (b) serum IL-6 level. Results are expressed as mean (SD), 8 rats per group. ^*^ *P* < 0.05 versus SO group; ^#^ *P* < 0.05 versus respective SAP group.

Serum concentrations of proinflammatory cytokines were analyzed to obverse anti-inflammatory process of hydrogen-rich saline after SAP. As illustrated in Figures 6(a) and 6(b), with the progress of SAP, serum levels of IL-1*β* and IL-6 increased continuously. However, there were significant decreases in HRS group compared to SAP group (*P* < 0.05).

### 3.6. Hydrogen-Rich Saline Attenuates the Inflammatory Response by Suppressing NF-*κ*B p65, TNF-*α*, and HMGB1 and Activating IL-10

**Figure 7:**
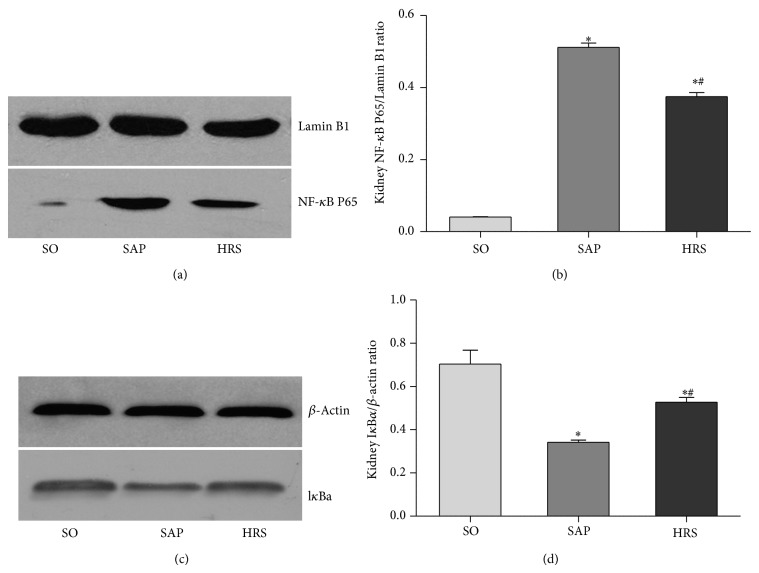
Western blotting for measuring the activation of NF-*κ*B p65. (a) Level of NF-*κ*B p65 in renal nucleoprotein at 12 hours, (b) densitometric analysis of the bands for the expression of NF-*κ*B p65/Lamin B in renal nucleoprotein, (c) level of I*κ*B in renal cytoplasmic protein at 12 hours, and (d) densitometric analysis of the bands for the ratio of I*κ*B/*β*-actin in renal nucleoprotein. Results are expressed as mean (SD), 8 rats per group. ^*^ *P* < 0.05 versus respective SO group; ^#^ *P* < 0.05 versus respective SAP group.

**Figure 8:**
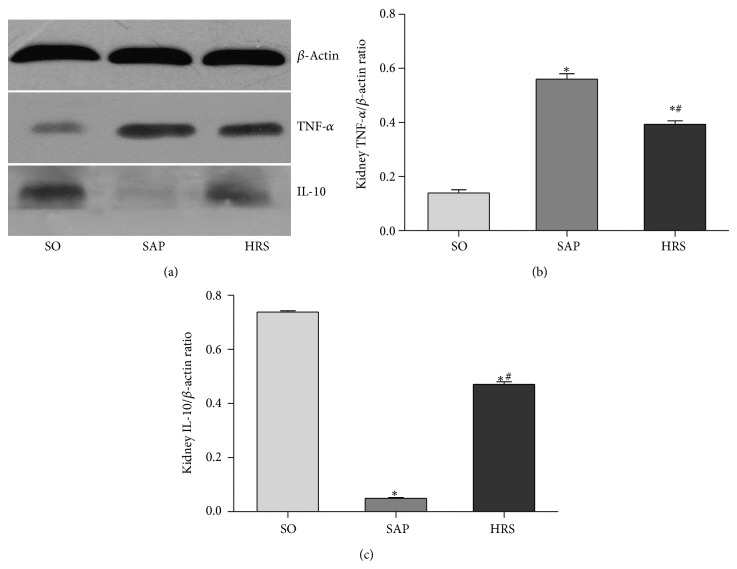
Western blotting for measuring the expression of TNF-*α* and IL-10 by using extract of renal total proteins at 12 hours. (a) Levels of TNF-*α* and IL-10 in renal total proteins. (b) and (c) Densitometric analysis of the bands for the ratios of TNF-*α*/*β*-actin and IL-10/*β*-actin. Results are expressed as mean (SD), 8 rats per group. ^*^ *P* < 0.05 versus respective SO group; ^#^ *P* < 0.05 versus respective SAP group.

**Figure 9:**
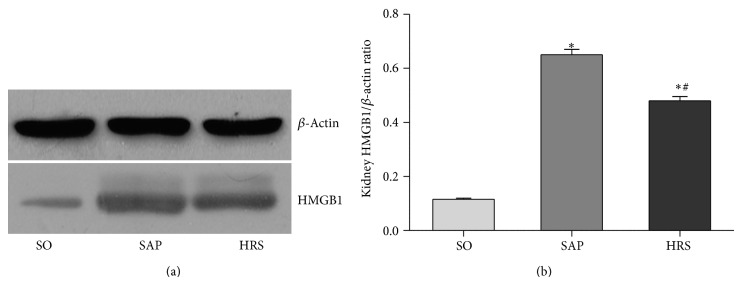
Western blotting for measuring the expression of HMGB1 by using extract of renal total proteins at 24 hours. (a) Level of HMGB1 and (b) densitometric analysis of the bands for the ratio of HMGB1/*β*-actin. Results are expressed as mean (SD), 8 rats per group. ^*^ *P* < 0.05 versus respective SO group; ^#^ *P* < 0.05 versus respective SAP group.

To obtain a mechanistic insight into the effects of hydrogen-rich saline used here, a Western blot analysis of the expression of NF-*κ*B p65 protein in the nucleus (Figure 7(a)) and I*κ*B*α* protein in the cytoplasm (Figure 7(c)). In addition, the expression of TNF-*α* (Figure 8(a)), IL-10 (Figure 8(a)), and HMGB1 (Figure 9(a)) was carried out, as well as densitometric analysis of the bands for the expression of these proteins ratio. As shown in Figures 7(b), 8(b), and 9(b), the ratios of the expression of NF-*κ*B p65 and TNF-*α* in renal tissues at 12 hours and HMGB1 at 24 hours were significantly decreased in HRS group when compared with SAP group (*P* < 0.05). As shown in Figures 7(d) and 8(c), the expression of I*κ*B and IL-10 in renal tissues at 12 hours was significantly decreased after SAP (*P* < 0.05), but the hydrogen-rich saline treatment induced a significant increase (*P* < 0.05).

## 4. Discussion

**Figure 10:**
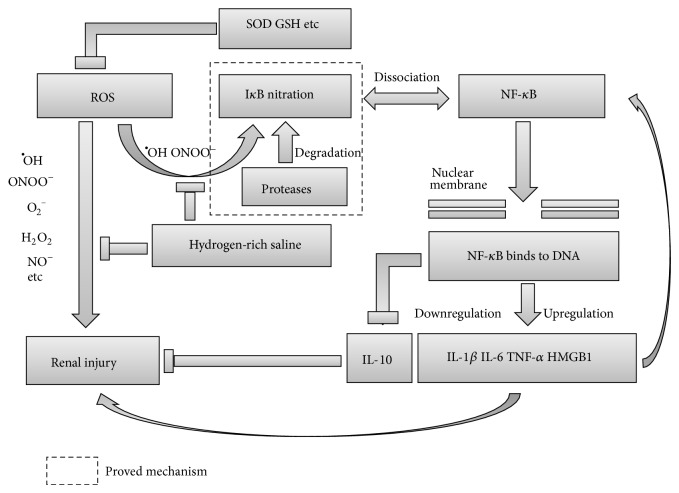
The protective mechanisms involved in the inflammatory response of renal tissues during severe acute pancreatitis. Hydrogen-rich saline protects the acute renal injury by antioxidant effect directly and by scavenging the overproduced ^•^OH and ONOO^−^ to inhibit the activation of NF-*κ*B.

ROS, which contain ^•^OH, O_2_, H_2_O_2_, ONOO, NO^−^, and so forth, are important cytotoxic molecules and signal mediators in the pathophysiological mechanisms of inflammatory diseases (Figure 10) [19, 20]. Among them, ^•^OH and ONOO^−^ are much more reactive than others and are regarded as major cytotoxic mediators of cellular oxidative damage [20]. A previous study had demonstrated that H_2_ reacts only with the strongest oxidants (^•^OH and ONOO^−^) and does not disrupt metabolic redox reactions or ROS concerned in cell signaling [6]. In addition, some studies also demonstrated that hydrogen-rich saline showed promising efficacy in animal models of several inflammatory diseases including AP [13–15], but there are no reports of hydrogen-rich saline in treating SAP associated acute kidney injury and other extrapancreatic organs injury. Therefore, our hypothesis is that hydrogen-rich saline may attenuate acute renal injury in SAP. In this study, we demonstrated (1) the renal injuries that were caused by the STC-induced acute pancreatitis were significantly improved by hydrogen-rich saline; (2) the mechanism by which hydrogen-rich saline attenuates acute renal injury was to reduce oxidative stress and inhibit activation of NF-*κ*B by inhibiting I*κ*B nitration and degradation; (3) hydrogen-rich saline downregulated the expression of “evil” inflammatory mediators and promote “angelic” inflammatory cytokine to produce.

In our experiments, conspicuous hyperamylasemia, hyperlipasemia, and pathological evidences like pancreatic hemorrhage and necrosis were observed in the STC-induced SAP model group. These results showed that the SAP model was successfully induced. The levels of serum Cr and BUN combined with aggravating morphological changes of the kidney showed obvious renal dysfunction and tissue injuries during the progression of SAP. We demonstrated hydrogen-rich saline treatment improves renal dysfunction and pancreatic and renal pathological injuries and inhibits inflammatory cytokines and oxidative stress. All of these observations indicate that hydrogen-rich saline exerts potent antioxidative and anti-inflammatory effects and attenuates the severity of SAP associated kidney injury in rats.

A growing number of reports have demonstrated that oxidative stress and its resultant production of ROS play prominent roles in the mechanism of inflammatory responses during AP [21, 22]. Under physiological condition, tissues contain various endogenous antioxidant enzymes like GSH and SOD, which scavenge ROS and prevent lipid peroxidation [23]. In the process of SAP, ROS were overproduced and then induced an imbalance between the ROS and endogenous antioxidants or antioxidant enzymes. The overproduced ROS was able to directly or indirectly damage the renal tissues and result in renal dysfunction and histological changes. Our results showed that the activity of SOD was significantly increased when using the hydrogen-rich saline treatment in SAP rat. Oppositely, the level of MDA was decreased. These results combined with the morphologic change of kidneys indicate that hydrogen-rich saline neutralizes the ROS and eases the renal oxidative damage.

Oxidative stress activates various cell signaling pathways [24–26], among which we focused particularly on NF-*κ*B. Blocking I*κ*B degradation can inhibit the activation of NF-*κ*B [27]. Thus, inhibiting I*κ*B degradation can attenuate the severity of experimental acute pancreatitis [28, 29]. In this study, we found that the translocation of activated NF-*κ*B into the nucleus was significantly increased, and the level of I*κ*B in cytoplasm was the lowest in SAP rat kidneys. But in using hydrogen-rich saline treated SAP rats, the activated NF-*κ*B in nucleus was decreased and the level of I*κ*B in cytoplasm was increased. These results strengthen the idea that the activation of NF-*κ*B could be inhibited by hydrogen-rich saline via blocking I*κ*B degradation. Available studies showed that the activation of NF-*κ*B was closely related to I*κ*B nitration [30]; I*κ*B molecules containing nitrated tyrosine residues may by themselves be targets for degradation by proteases, leading to NF-*κ*B activation [30, 31], and it is necessary that ROS (^•^OH and ONOO^−^) be involved in the process of I*κ*B nitrated reaction [32]. In our experiments, the expression of 3-nitrotyrosine had a significant increase after SAP, but the expression was inhibited by hydrogen-rich saline. Therefore, we conclude that hydrogen-rich saline affects the formation of I*κ*B nitrated tyrosine residues by scavenging the overproduced ROS (^•^OH and ONOO^−^), thus inhibiting the activation of NF-*κ*B.

Cytokines such as TNF-*α*, IL-1*β*, and IL-6 play a pivotal role in SAP and exert a major influence on the outcome of the disease, in particular by triggering the systemic inflammatory response and multisystem organ failure, the latter being a marked feature of SAP and responsible for most of the associated mortality [33, 34]. Moreover, the “angelic” inflammatory cytokine such as IL-10 might play an important role in the process of the disease. Available studies demonstrated that IL-10 could inhibit the production of TNF-*α*, IL-1*β*, and IL-6 [35] and take part in negative feedback that controls acute inflammatory response [36]. Our results showed that the levels of IL-1*β* and IL-6 in serum and TNF-*α* in renal tissue were significantly reduced in using hydrogen-rich saline treated rats when compared with SAP ones. In addition, the level of IL-10 was elevated. These findings clarified that hydrogen-rich saline inhibits the production of these cytokines except IL-10 via reducing oxidative stress and inhibiting NF-*κ*B activation.

High-mobility group box protein 1 (HMGB1) is a late activator in the inflammatory cascade. It has the capacity to induce cytokines releasing and activate inflammatory cells after being translocated into extracellular space [37]. It is reported that cerulein-induced HMGB1 expression was reduced in pancreatic acinar cells by inhibiting NF-*κ*B activation [38]. Our results concerning HMGB1 expression at 24 hours were in agreement with other reports and strengthen the idea that the expression of HMGB1 is inhibited by hydrogen-rich saline via inhibiting NF-*κ*B activation.

Myeloperoxidase (MPO) is a biochemical marker for neutrophil infiltration in studies of multiple-organs injury in AP, and its activity in kidney related to the severity of renal injury [39, 40]. Our results showed that the increase of renal MPO and function parameters indicates the progressive aggravation of SAP associated renal injury. The hydrogen-rich saline treatment significantly reduced these outcomes and suggested that it could attenuate the renal neutrophil infiltration.

In conclusion, our study demonstrated that hydrogen-rich saline could attenuate acute renal injury in sodium taurocholate-induced SAP rat model. By inhibiting NF-*κ*B activation and scavenging ROS, hydrogen-rich saline prevented the development of inflammatory cascade and eased renal oxidative damage. Thus, we presented the novel antioxidant hydrogen-rich saline, which is easier and safer to apply, could reduce ROS and suppress inflammatory mediators, and may have the potential application to alleviate SAP associated acute renal injury clinically.
